# The cochaperone BAG3 promotes the stabilization of p53 under heat stress conditions

**DOI:** 10.1002/2211-5463.70096

**Published:** 2025-07-31

**Authors:** Ngoc Nguyen Thi Minh, Esther Lee, Soo‐A Kim

**Affiliations:** ^1^ Department of Biochemistry Dongguk University College of Oriental Medicine Gyeongju Korea

**Keywords:** BAG3, heat stress, Hsp70, p53

## Abstract

Bcl‐2‐associated athanogene 3 (BAG3) is the only member of the BAG cochaperone family that is induced by stressful stimuli such as heat shock and heavy metals. In the present study, *bag3* knockout (KO) HeLa cells were generated via the CRISPR‐Cas9 system, and the role of BAG3 in relation to p53 under heat stress conditions was investigated. Normally, the levels of p53 were low in both wild‐type (WT) and KO cells, while heat shock increased the levels of nuclear p53 in both cell lines. However, the increased level of p53 was much greater in WT cells than in KO cells, which suggested that BAG3 played a role in controlling the level of p53 under heat stress conditions. The mRNA level of p53 did not increase in either WT or KO cells during the heat stress period, which suggested that the differences in the levels of p53 were not due to transcriptional regulation. Both treatment with the proteasome inhibitor MG132 and heat shock drastically increased p53 levels to a similar extent in WT cells. Interestingly, both BAG3 and Hsp70 rapidly translocated to the nucleus and formed a complex with p53 upon heat stress. During a 1‐h recovery period from heat stress, the transcriptional activity of p53 increased up to 4‐fold in WT cells, but only 1.69‐fold in KO cells. These results demonstrate that Hsp70 and BAG3 are involved in the quality control of p53 under heat stress conditions and suggest a role for BAG3 as a cochaperone protein.

## Graphical abstract

Under heat stress, BAG3 translocates to the nucleus and forms a complex with Hsp70 and p53, thereby promoting p53 stabilization and enhancing its transcriptional activity. These findings suggest that BAG3 functions as a cochaperone that supports p53‐mediated stress responses in cooperation with Hsp70.

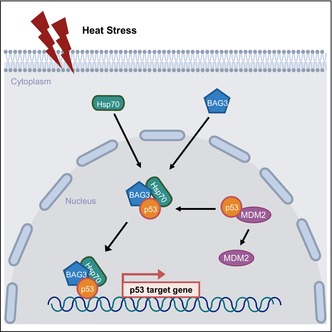

**Abbreviations**
BAG3: Bcl‐2 associated athanogene 3
Cas9: CRISPR‐associated protein 9
CRISPR: clustered regularly interspaced short palindromic repeats
EGFP: enhanced green fluorescent protein
FITC: fluorescein isothiocyanate
HSP: heat shock protein

Cells are continuously exposed to a variety of stressful conditions, such as heat, heavy metals, ionizing radiation, and ultraviolet radiation. Heat stress induces the misfolding or unfolding of proteins, resulting in the disruption of essential cellular signaling pathways [1]. Cells respond to heat stress via a cellular defense mechanism by activating and inducing highly conserved heat shock proteins (Hsps), which function as molecular chaperones. Hsp70 is a major stress‐inducible molecular chaperone that plays a critical role in various protein folding processes. In addition to participating in the folding of newly synthesized proteins, Hsp70 plays an important role in maintaining cellular protein homeostasis by refolding damaged proteins and preventing native proteins from unfolding under stress conditions [2, 3].

Cochaperones are components of the protein quality control machinery, and they are required for nucleotide exchange and to form complexes with other partner proteins [4, 5]. The Bcl‐2‐associated athanogene (BAG) cochaperone family consists of six family members (BAG1–6). All members of this family contain a BAG domain, through which they bind to Hsp70 and regulate its activity [6, 7]. Among BAG proteins, BAG3 is the most attractive target because it is the only member that can be induced by stressful stimuli such as heat shock, heavy metal exposure, and oxidative stress [8, 9]. BAG3 contains additional modular protein domains, such as the WW domain, two IPV motifs, and a proline‐rich region (PXXP). Through these domains, BAG3 can interact with HspB6, HspB8, dynein, and phospholipase C (PLC)‐γ and is implicated in various cellular processes, including macroautophagy, proliferation, metastasis, apoptosis, and proteasomal degradation [7, 10, 11, 12, 13].

p53 is a well‐documented tumor suppressor, and ~ 50–60% of human cancers carry homozygous mutations in the *p53* gene [14]. Typically, the intracellular level of p53 is very low because of its short half‐life (approximately 20 min). The level of p53 is regulated mainly through posttranslational modifications. The MDM2 protein binds to the amino terminus of p53, leading to its degradation through the ubiquitin‐proteasome‐mediated pathway [15]. Various cellular stresses, such as ionizing radiation, ultraviolet light, hypoxia, and heat shock, induce the accumulation of p53 in the nucleus [15, 16, 17]. Interestingly, Marco and colleagues reported that p53 interacted with BAG3 [18]. Considering the possible role of BAG3 as a cochaperone, investigations into the relationship between BAG3 and p53 under heat stress conditions are warranted.

In this study, we generated *bag3* knockout (KO) cells using the CRISPR/Cas9 genome‐editing system and demonstrated the role of BAG3 as a cochaperone involved in p53 protein stability under heat stress conditions.

## Materials and methods

### Cell culture and treatment

Human cervical carcinoma HeLa cells were purchased from American Type Culture Collection (Manassas, VA, USA). HeLa cells were cultured in DMEM supplemented with 10% FCS, 100 units·mL^−1^ penicillin, and 100 μg·mL^−1^ streptomycin (all from WELGENE, Gyeongsan‐si, Korea). The cells were cultured at 37 °C in a humidified atmosphere with 5% CO_2_. For heat shock treatment, 35 mm culture plates were wrapped with Parafilm and then immersed in a 42 °C water bath (WiseBath™ Fuzzy Control System, Daihan Scientific, Wonju‐si, Korea) for 1 h [19, 20].

### Generation of a *bag3* knockout cell line

The *bag3* knockout (KO) HeLa cells were generated using the CRISPR/Cas9‐mediated Guide‐it™ Complete sgRNA Screening System (Takara Bio USA, Inc., Mountain View, CA, USA) according to the manufacturer's instructions. Briefly, the following BAG3‐specific target sequence was designed via the CRISPR RGEN tools (http://rgenome.net): 5′‐CCCATGATGCAGGTGGCGTC‐3′. The sgRNA template was synthesized by PCR using the following forward primer: 5′‐CCTCTAATACGACTCACTATAGGCCCATGATGCAGGTGGCGTCGTTTAAGAGCTATGC‐3′.

The PCR product was used as a template for *in vitro* transcription to synthesize the sgRNA. The sgRNA was then mixed with recombinant Cas9 to form a ribonucleoprotein (RNP) complex. The Cas9‐sgRNA RNP complex was delivered into HeLa cells by electroporation using the Neon™ Transfection System (Thermo Fisher Scientific, Waltham, MA, USA). Electroporation was performed using the following parameters: 1005 V, 35 ms, 2 pulses. Following transfection, monoclonal cells were isolated by limiting dilution and expanded. Knockout efficiency was validated by western blot analysis using an anti‐BAG3 antibody (Abcam, Cambridge, UK).

### Immunoprecipitation and immunoblotting

For immunoprecipitation, HeLa cells were transiently transfected with FuGENE^®^ HD (Promega, Madison, WI, USA) according to the manufacturer's instructions. The following vector inserts were used: N‐terminal FLAG‐tagged full‐length BAG3 (BAG3‐WT, amino acids 1–575), ΔBAG (BAG3‐ΔBAG, amino acids 1–412), ΔWW/IPV (BAG3‐ΔWW/IPV, amino acids 101–575), ΔWW/2 IPV (BAG3‐ΔWW/2 IPV, amino acids 213–575), N‐terminal myc‐tagged full‐length p53 (p53‐WT, amino acids 1–393), ΔTET/REG (p53‐ΔTET/REG, amino acids 1–292), ΔTAD/REG (p53‐ΔTAD/REG, amino acids 98–362), ΔTAD (p53‐ΔTAD, amino acids 98–393), and N‐terminal EGFP‐tagged full‐length Hsp70 (Hsp70‐WT, amino acids 1–641). The delta (Δ) symbol indicates a deletion of a specific DNA sequence in the cloning vector. Forty hours after transfection, the cells were lysed with RIPA buffer (PBS supplemented with 1% Nonidet P‐40, 0.5% sodium deoxycholate, 1 mm PMSF, 1 μg·mL^−1^ aprotinin, and 1 mm sodium orthovanadate), after which immunoprecipitation was performed as described previously [19].

For immunoblotting, cytoplasmic and nuclear proteins were obtained by a previously described method [19]. The protein samples were resolved by SDS/polyacrylamide gel electrophoresis and immunoblotted with the following antibodies: anti‐p53 (sc‐126), anti‐Hsp70 (sc‐24), anti‐Hsp90 (sc‐13 119), anti‐FLAG (sc‐166 355), anti‐myc (sc‐40), and anti‐GFP (sc‐9996) from Santa Cruz Biotechnology (Dallas, TX, USA); anti‐BAG3 (ab47124) from Abcam; anti‐β‐actin (A1978) from Sigma‐Aldrich (St. Louis, MO, USA); and anti‐p21 (A1483) and anti‐Bax (A0207) from ABclonal Science (Woburn, MA, USA). The density of each band was quantified by using the imagej software (version 1.53k; NIH, Bethesda, MD, USA).

### Immunofluorescence analyses

HeLa cells were seeded on four‐chamber slides (Thermo Fisher Scientific) at a density of 5 × 10^4^ cells·mL^−1^. The cells were cultured overnight and heat stressed at 42 °C for 1 h, followed by recovery at 37 °C for 1 h if necessary. The cells were then washed twice with PBS and fixed with methanol for 2 min. Immunostaining was performed as described previously [19]. The secondary antibodies used for immunostaining were as follows: FITC‐conjugated anti‐mouse (Vector Laboratories, Burlingame, CA, USA), Alexa Fluor 594‐conjugated anti‐mouse (Thermo Fisher Scientific), and FITC‐conjugated anti‐rabbit (Vector Laboratories) antibodies. Nuclei were stained with 4′,6‐diamidino‐2‐phenylindole (DAPI; Thermo Fisher Scientific). Fluorescence analysis was performed under a Lion Heart FX automated microscope (Agilent, Santa Clara, CA, USA).

### RNA isolation and RT‐PCR

The cells were heat treated at 42 °C for 1 h, after which total RNA was extracted with TRI‐Solution™ (BSK Bio Science, Daegu, Korea) according to the manufacturer's instructions. Semiquantitative RT‐PCR was performed with a ONE‐STEP RT‐PCR premix kit (iNtRON, Gyeonggi‐do, Korea). The specific primers used for RT‐PCR were as follows: p53 forward (5′‐CTTTGAGGTGCGTGTTTGTGCC‐3′) and reverse (5′‐AATGGAAGTCCTGGGTGCTTCTG‐3′); and GAPDH forward (5′‐CCAAGGTCATCCATGACAACTTTG‐3′) and reverse (5′‐GTCATACCAGGAAATGAGCTTGACA‐3′) primers. RT‐PCR was conducted under the following conditions: 1 cycle of 30 min at 45 °C; 1 cycle of 5 min at 94 °C; 27 cycles of 30 s at 94 °C, 30 s at 56 °C, and 40 s at 72 °C; and a final extension at 72 °C for 5 min.

### p53 transcriptional activity assay

The transcriptional activity of p53 was measured with the TransAM™ p53 transcription factor assay kit according to the manufacturer's instructions (Active Motif, Carlsbad, CA, USA). Briefly, the nuclear fraction was purified and then added to a 96‐well plate in which p53‐specific double‐stranded oligonucleotides were immobilized. After 1 h of incubation at room temperature, an anti‐p53 antibody was added for 1 h, followed by incubation with an HRP‐conjugated anti‐rabbit secondary antibody. After 1 h, color was developed by using a development solution, and colorimetric changes were subsequently measured at 450 nm on an iMark™ microplate reader (Bio‐Rad, Hercules, CA, USA).

### Cell proliferation assay

The cells were seeded on 12‐well plates at a density of 5 × 10^4^ cells·mL^−1^. After overnight incubation, the cells were subjected to heat treatment at 42 °C for 1 h, followed by recovery at 37 °C for the indicated time periods. Cell proliferation was assessed using a 3‐(4,5‐dimethylthiazol‐2‐yl)‐2,5‐diphenyltetrazolium bromide (MTT) assay, as previously described [21].

### Statistical analysis

The data are expressed as the means ± SD. Differences between groups were analyzed by Student's *t*‐test and ANOVA (GraphPad Prism, San Diego, CA, USA) followed by Dunnett's *post hoc* test. *P* < 0.01 was considered to indicate statistical significance. The ‘*’ sign indicates *P* < 0.01.

## Results

### BAG3 affects the level of p53 upon heat stress

**Fig. 1:**
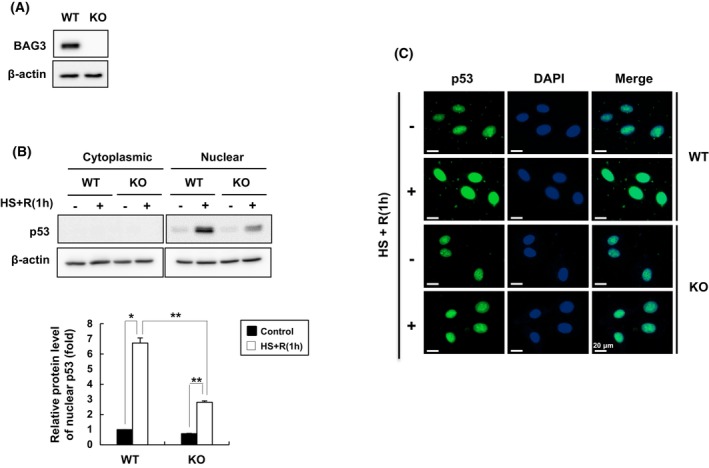
BAG3 affects the level of heat‐induced nuclear p53 in HeLa cells. (A) *Bag3* gene KO HeLa cells were generated via the CRISPR/Cas9 system. Total cell lysates were obtained, and the expression levels of BAG3 were determined by western blot analysis. (B, C) WT and *bag3* KO HeLa cells were subjected to heat shock at 42 °C for 1 h, followed by recovery at 37 °C for 1 h. (B) Cytoplasmic and nuclear fractions were isolated, and p53 levels were determined by western blot analysis. Actin was used as a loading control. The band densities were quantified by densitometry. The data are expressed as the means ± SD of three independent experiments. Statistical significance was determined by Student's *t*‐test or ANOVA with Dunnett's *post hoc* test. *P < 0.01, **P < 0.02, as indicated. (C) Immunostaining was performed using a primary antibody against p53 and a FITC‐conjugated secondary antibody. DAPI was used for nuclear staining. The subcellular localization of p53 was observed by fluorescence microscopy. Scale bar: 20 μm. All experiments were performed independently at least three times.

To assess the role of BAG3 under stress conditions, *bag3* gene KO clones were generated from HeLa cells using the CRISPR/Cas9 system (Fig. 1A). Parental (WT) and *bag3* KO cells were subjected to heat shock to investigate the effects of BAG3 expression on the p53 level. Interestingly, when the cells were subjected to heat stress at 42 °C for 1 h and allowed to recover at 37 °C for 1 h, the level of nuclear p53 dramatically increased in the WT cells compared with that in the *bag3* KO cells (Fig. 1B). Immunostaining confirmed these results and showed that under control condition, p53 levels were low and mainly localized in the nucleus. Compared with that in *bag3* KO cells, the level of nuclear p53 in WT cells largely increased under heat stress conditions (Fig. 1C). These results suggest that heat stress increases the level of nuclear p53 and that BAG3 may play an important role in this increase.

### BAG3 affects p53 protein stability

**Fig. 2:**
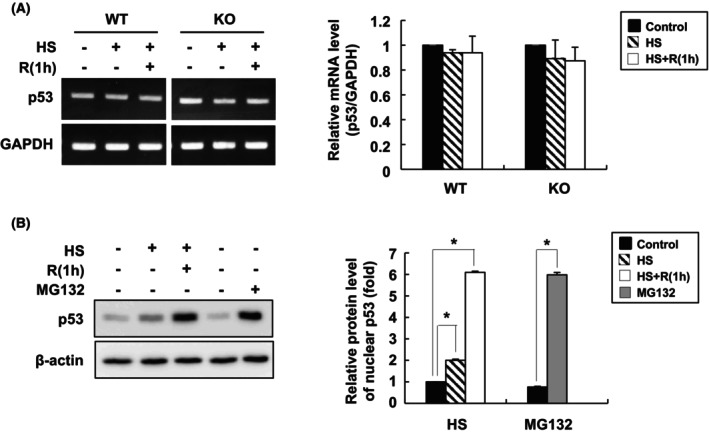
BAG3 is involved in the stabilization of p53 under heat stress conditions. (A) WT and KO HeLa cells were subjected to heat shock at 42 °C for 1 h, followed by recovery at 37 °C for 1 h. Total RNA was purified, and semiquantitative RT‐PCR was performed. GAPDH was used as an internal control. The band densities were quantified by densitometric analysis, and the results are presented in the bar graph on the right. The data are expressed as the means ± SD of three independent experiments. No statistically significant differences were observed. (B) WT cells were treated with MG132 (1 μm) for 24 h. Nuclear proteins were purified, and the levels of p53 were determined by western blot analysis. The band densities were quantified by densitometry. The data are expressed as the means ± SD of three independent experiments. Statistical significance was performed using Student's *t*‐test or ANOVA followed by Dunnett's test. *P < 0.01.

To understand the mechanism by which p53 is induced upon heat stress, the mRNA levels of p53 were examined via semiquantitative RT‐PCR. As shown in Fig. 2A, the levels of p53 mRNA did not change following heat stress or during the recovery period in either WT or KO cells, which suggested that the heat stress‐induced increases in the p53 protein levels were not due to increased transcription of the gene. The levels of p53 are normally regulated by posttranslational modifications. Through the ubiquitin‐proteasome‐mediated pathway, p53 is rapidly degraded, which results in its short half‐life of approximately 5–20 min, depending on the cell type. Interestingly, when the cells were treated with MG132, a proteasome inhibitor, p53 levels dramatically increased, up to 6.0‐fold, which was comparable to the 6.1‐fold increase observed under heat stress followed by recovery (Fig. 2B).

### BAG3 and Hsp70 translocate to the nucleus upon heat stress

**Fig. 3:**
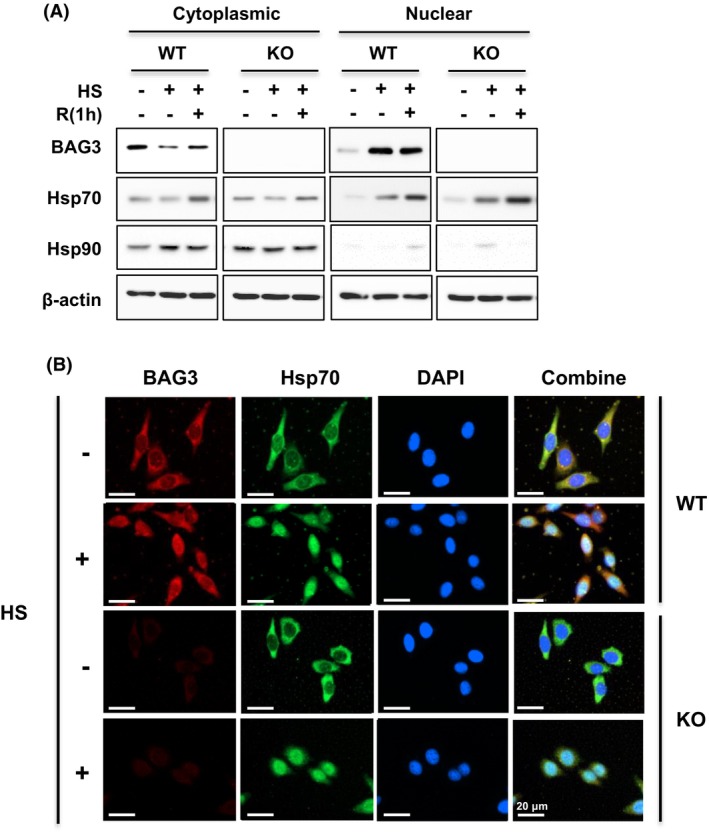
BAG3 and Hsp70 translocate to the nucleus upon heat stress. (A) WT and KO HeLa cells were subjected to heat shock at 42 °C for 1 h, followed by recovery at 37 °C for 1 h. Cytoplasmic and nuclear fractions were isolated, and the levels of BAG3, Hsp70, and Hsp90 were determined by western blot analysis. (B) WT and KO HeLa cells were subjected to heat shock at 42 °C for 1 h. For immunostaining, the cells were incubated with primary antibodies against BAG3 or Hsp70 and then incubated with Texas Red‐ or FITC‐conjugated secondary antibodies, respectively. The nuclei were stained with DAPI. The subcellular localization of BAG3 and Hsp70 was observed by fluorescence microscopy. Scale bar: 20 μm. All experiments were performed independently at least three times.

To investigate the effect of BAG3 on the level of p53, WT and *bag3* KO cells were heat treated at 42 °C for 1 h and then allowed to recover at 37 °C for 1 h. Cytoplasmic and nuclear protein fractions were isolated, and the levels of BAG3 and Hsp70 were examined. As shown in Fig. 3A, BAG3 was localized mainly to the cytoplasm. Upon heat stress, BAG3 rapidly translocated to the nucleus in WT cells. Hsp70 was also localized to the cytoplasm, and heat stress induced its nuclear translocation. The presence of BAG3 did not affect the nuclear translocation of Hsp70 (Fig. 3A). Unlike BAG3 and Hsp70, Hsp90 did not translocate to the nucleus under heat stress conditions, suggesting that nuclear translocation under heat stress is specific to BAG3 and Hsp70. Immunostaining confirmed the translocation of BAG3 to the nucleus upon heat stress. Regardless of the presence of BAG3, Hsp70 always translocated to the nucleus upon heat stress (Fig. 3B).

### BAG3 and Hsp70 interact with p53 in the nucleus

**Fig. 4:**
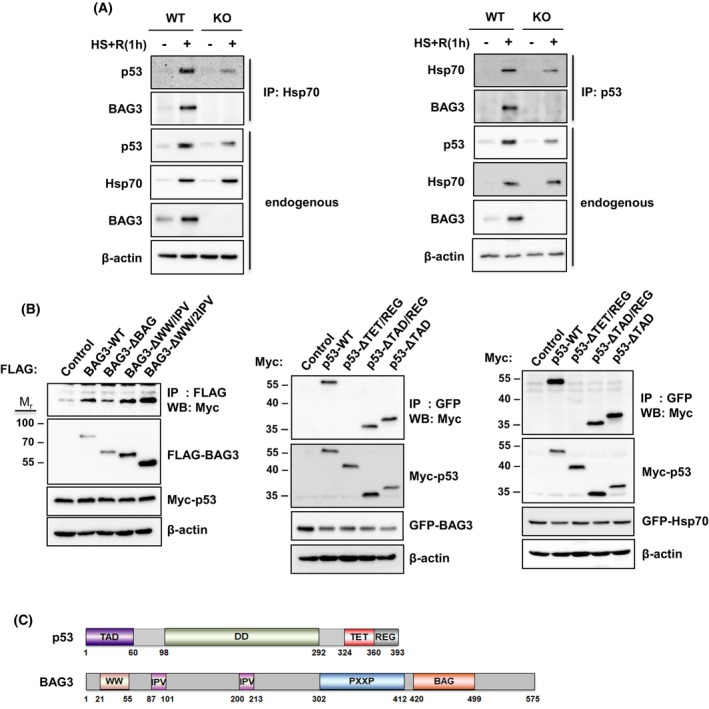
BAG3, Hsp70, and p53 form a complex in the nucleus. (A) HeLa cells were subjected to heat stress at 42 °C for 1 h, followed by recovery at 37 °C for 1 h. Nuclear fractions were prepared, and immunoprecipitation was performed by using anti‐Hsp70 or anti‐p53 antibodies. Coprecipitated p53 and BAG3 (left panel) or Hsp70 and BAG3 (right panel) were analyzed by western blot analysis. Endogenous protein levels were monitored by western blot analysis. (B) HeLa cells were cotransfected with both FLAG‐tagged BAG and Myc‐tagged p53 vectors (left panel), GFP‐tagged BAG and Myc‐tagged p53 vectors (middle panel) or GFP‐tagged Hsp70 and Myc‐tagged p53 vectors (right panel). After 30 h, total cell extracts were prepared, and an immunoprecipitation assay was performed with anti‐FLAG or anti‐GFP antibodies. (C) Schematic diagrams of p53 and BAG3 domain organization. Domain boundaries were obtained from the SMART program.

We next investigated whether heat stress induces the formation of BAG3, Hsp70, and p53 complexes in the nucleus. Following heat stress and recovery, BAG3 WT and KO cells were lysed, and an immunoprecipitation assay was performed. As shown in Fig. 4A, both p53 and BAG3 coimmunoprecipitated with Hsp70 in WT cells. The same results were obtained when Hsp70 or BAG3 was pulled down with p53. Although the amount was low, p53 and Hsp70 complexes were also detected in *bag3* KO cells.

To further characterize the interactions among BAG3, p53, and Hsp70, FLAG‐tagged BAG3 and its deletion mutant constructs were cotransfected with a Myc‐tagged p53 construct into HeLa cells. An immunoprecipitation assay revealed that p53 interacted with BAG3 through the BAG domain (Fig. 4B, left panel). Similarly, cells transfected with Myc‐tagged p53 and its deletion constructs, together with GFP‐tagged BAG3 or GFP‐tagged Hsp70 constructs, showed that both BAG3 and Hsp70 interacted with p53 through the TET domain (Fig. 4B, middle and right panels). The domain organizations of p53 and BAG3 are presented in Fig. 4C.

### BAG3 plays a role in p53 transcriptional activity

**Fig. 5:**
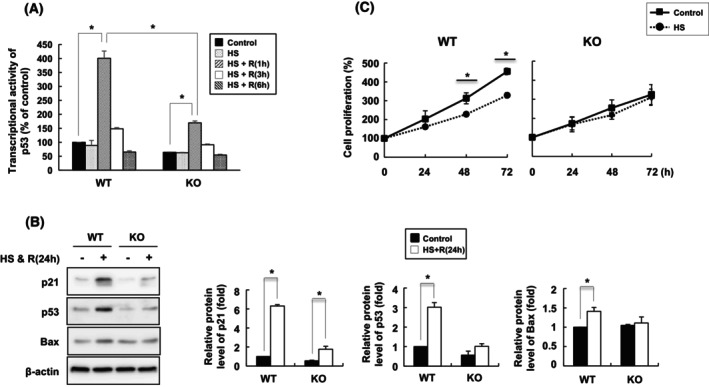
BAG3 plays a role in the transcriptional activity of p53. (A) HeLa cells were heat stressed at 42 °C for 1 h, followed by recovery at 37 °C for 1, 3, or 6 h. Nuclear fractions were prepared, and the transcriptional activity of p53 was measured. The data are expressed as the means ± SD of three independent experiments. Statistical significance was determined by Student's *t*‐test or ANOVA with Dunnett's *post hoc* test. *P < 0.01. (B) The cells were subjected to heat shock for 1 h, followed by recovery for 24 h. Total cell lysates were prepared, and the expression levels of p53 target genes were examined by western blot analysis. Densitometric analysis of the western blot bands was performed, and the quantification results are presented in the bar graph on the right. The data are expressed as the means ± SD of three independent experiments. *P < 0.01. (C) The cells were subjected to heat shock for 1 h, followed by recovery for 24, 48, or 72 h. Cell proliferation was assessed using the MTT assay. The data are presented as the means ± SD of three independent experiments. *P < 0.01.

Because BAG3 and p53 form a complex upon heat stress, we next investigated whether BAG3 can affect the transcriptional activity of p53. For this experiment, the cells were heat stressed for 1 h and then allowed to recover for the indicated periods. In BAG3 WT cells, the transcriptional activity of p53 largely increased, up to 4‐fold, during the 1 h recovery period following heat stress (Fig. 5A). However, in BAG3 KO cells, p53 transcriptional activity increased only 1.69‐fold during the same recovery period. Consistent with these observations, both p21, a well‐known p53 target gene, and p53 itself were strongly induced in WT cells 24 h after heat stress, suggesting that BAG3 plays a role in inducing p53 transcriptional activity under heat stress conditions (Fig. 5B). Furthermore, a cell proliferation assay demonstrated that heat stress triggered cell cycle arrest in WT cells, whereas BAG3 KO cells did not exhibit heat stress‐induced cell cycle arrest (Fig. 5C).

## Discussion

The heat shock response is an essential defense mechanism that protects cells from various harmful conditions. In response to heat stress, cells induce the activation and synthesis of Hsp70, a molecular chaperone that prevents the misfolding or aggregation of damaged proteins and facilitates protein refolding or degradation [2, 3, 22]. Previously, we demonstrated that the cochaperone BAG3 rapidly accumulated in the nucleus upon heat stress and suggested that it functioned as a regulator of the heat shock response [19]. Interestingly, several studies have shown that heat stress can also induce the accumulation of the tumor suppressor p53 [15, 16, 23]. However, the relationship between BAG3 and p53 under stress conditions remains unclear.

As a cochaperone, BAG3 directly interacts with Hsp70 and plays a role in Hsp70 activity in protein quality control. To facilitate protein folding, Hsp70 functions through an ATPase cycle, during which Hsp70 can alternate between its ATP/ADP‐binding states, which enable it to interact with and release partner proteins. Therefore, cochaperones such as BAG3 are required for nucleotide exchange or binding with partner proteins [5]. Doong and colleagues showed that through interaction with Hsp70, BAG3 inhibited the degradation of Hsp70 partner proteins and led to the accumulation of polyubiquitinated Akt [24]. By using *bag3* KO HeLa cells, we demonstrated that the heat stress‐induced accumulation of p53 in the nucleus was strongly affected by the expression of BAG3 (Fig. 1). Under the same conditions, both BAG3 and Hsp70 translocated to the nucleus, where they formed a complex with p53 (Figs 3 and 4). More importantly, the transcriptional activity of p53 was also strongly affected by BAG3 during heat stress and the subsequent recovery period (Fig. 5). These results strongly demonstrate that BAG3 plays a role in protein stability and the subsequent transcriptional activity of p53 under heat stress conditions.

Notably, regardless of the presence of BAG3, Hsp70 translocated to the nucleus and formed a complex with p53 upon heat stress (Figs 3 and 4). However, the heat stress‐induced protein level and subsequent transcriptional activity of p53 in BAG3 KO cells were markedly lower than those in BAG3 WT cells. As BAG3 has been referred to as a ‘cochaperone’ that assists chaperone function, its absence did not have a dramatic effect on cell fate. Nevertheless, our data clearly demonstrate that BAG3 acts as a functional chaperone for Hsp70, enabling it to execute its role effectively.

Previous studies have shown that heat stress induces the accumulation of p53 and cell cycle arrest in a p53‐dependent manner [23, 25, 26]. However, the precise molecular mechanisms are not fully understood. Herein, we first demonstrated the role of BAG3 in p53 stability following heat stress. Although this study was conducted using only HeLa cells, an immortalized cervical cancer cell line, additional validation in other cell types would strengthen the generalizability of BAG3's functional impact on p53 regulation under stress conditions. Considering the importance of p53 in cancer progression, our results suggest that Hsp70 and its cochaperone BAG3 are powerful targets for cancer treatment.

## Conflict of interest

The authors declare no conflict of interest.

## Author contributions

NNTM and EL performed the experiments and analyzed the data; S‐AK and NNTM wrote the manuscript; S‐AK designed the experiments and supervised the study.

## References

[1] Richter K , Haslbeck M and Buchner J (2010) The heat shock response: life on the verge of death. Mol Cell 40, 253–266. 20965420 10.1016/j.molcel.2010.10.006

[2] Kampinga HH and Craig EA (2010) The HSP70 chaperone machinery: J proteins as drivers of functional specificity. Nat Rev Mol Cell Biol 11, 579–592. 20651708 10.1038/nrm2941 PMC3003299

[3] Hartl FU , Bracher A and Hayer‐Hartl M (2011) Molecular chaperones in protein folding and proteostasis. Nature 475, 324–332. 21776078 10.1038/nature10317

[4] Genest O , Wickner S and Doyle SM (2019) Hsp90 and Hsp70 chaperones: collaborators in protein remodeling. J Biol Chem 294, 2109–2120. 30401745 10.1074/jbc.REV118.002806 PMC6369297

[5] Faust O and Rosenzweig R (2020) Structural and biochemical properties of Hsp40/Hsp70 chaperone system. In HSF1 and Molecular Chaperones in Biology and Cancer ( Mendillo ML , Pincus D and Scherz‐Shouval R , eds), pp. 3–20. Springer, Switzerland. 10.1007/978-3-030-40204-4_1 32297208

[6] Rosati A , Graziano V , De Laurenzi V , Pascale M and Turco MC (2011) BAG3: a multifaceted protein that regulates major cell pathways. Cell Death Dis 2, e141. 21472004 10.1038/cddis.2011.24 PMC3122056

[7] Behl C (2016) Breaking BAG: the co‐chaperone BAG3 in health and disease. Trends Pharmacol Sci 37, 672–688. 27162137 10.1016/j.tips.2016.04.007

[8] Pagliuca MG , Lerose R , Cigliano S and Leone A (2003) Regulation by heavy metals and temperature of the human BAG‐3 gene, a modulator of Hsp70 activity. FEBS Lett 541, 11–15. 12706811 10.1016/s0014-5793(03)00274-6

[9] Rosati A , Di Salle E , Luberto L , Quinto I , Scala G , Turco MC and Pascale M (2009) Identification of a Btk‐BAG3 complex induced by oxidative stress. Leukemia 23, 823–824. 19212330 10.1038/leu.2009.23

[10] Doong H , Price J , Kim YS , Gasbarre C , Probst J , Liotta LA , Blanchette J , Rizzo K and Kohn E (2000) CAIR‐1/BAG‐3 forms an EGF‐regulated ternary complex with phospholipase C‐gamma and Hsp70/Hsc70. Oncogene 19, 4385–4395. 10980614 10.1038/sj.onc.1203797

[11] Zhou J , Chow HM , Liu Y , Wu D , Shi M , Li J , Wen L , Gao Y , Chen G , Zhuang K *et al*. (2020) Cyclin‐dependent kinase 5‐dependent BAG3 degradation modulates synaptic protein turnover. Biol Psychiatry 87, 756–769. 31955914 10.1016/j.biopsych.2019.11.013

[12] Behl C (2011) BAG3 and friends: co‐chaperones in selective autophagy during aging and disease. Autophagy 7, 795–798. 21681022 10.4161/auto.7.7.15844

[13] Shi H , Xu H , Li Z , Zhen Y , Wang B , Huo S , Xiao R and Xu Z (2016) BAG3 regulates cell proliferation, migration, and invasion in human colorectal cancer. Tumour Biol 37, 5591–5597. 26577854 10.1007/s13277-015-4403-1

[14] Baugh EH , Ke H , Levine AJ , Bonneau RA and Chan CS (2018) Why are there hotspot mutations in the TP53 gene in human cancers? Cell Death Differ 25, 154–160. 29099487 10.1038/cdd.2017.180 PMC5729536

[15] Oren M (1999) Regulation of the p53 tumor suppressor protein. J Biol Chem 274, 36031–36034. 10593882 10.1074/jbc.274.51.36031

[16] Nitta M , Okamura H , Aizawa S and Yamaizumi M (1997) Heat shock induces transient p53‐dependent cell cycle arrest at G1/S. Oncogene 15, 561–568. 9247309 10.1038/sj.onc.1201210

[17] Lee YK , Heo HH , Kim N , Park UH , Youn H , Moon EY , Kim EJ and Um SJ (2024) Tumor antigen PRAME is a potential therapeutic target of p53 activation in melanoma cells. BMB Rep 57, 299–304. 38835116 10.5483/BMBRep.2023-0246 PMC11214892

[18] De Marco M , Troisi J , Giugliano L , Rosati A , D'Antonio A , Iaccarino R , Capunzo M , Salzano F , Martinelli R , Cavallo P *et al*. (2020) BAG3 interacts with p53 in endometrial carcinoma. Cell Oncol 43, 957–960. 10.1007/s13402-020-00543-3 PMC12990752 32578139

[19] Jin YH , Ahn SG and Kim SA (2015) BAG3 affects the nucleocytoplasmic shuttling of HSF1 upon heat stress. Biochem Biophys Res Commun 464, 561–567. 26159920 10.1016/j.bbrc.2015.07.006

[20] Lang BJ , Nguyen L , Nguyen HC , Vieusseux JL , Chai RCC , Christophi C , Fifis T , Kouspou MM and Price JT (2012) Heat stress induces epithelial plasticity and cell migration independent of heat shock factor 1. Cell Stress Chaperones 17, 765–778. 22791010 10.1007/s12192-012-0349-z PMC3468677

[21] Kim SA , Kim YC , Kim SW , Lee SH , Min JJ , Ahn SG and Yoon JH (2007) Antitumor activity of novel indirubin derivatives in rat tumor model. Clin Cancer Res 13, 253–259. 17200363 10.1158/1078-0432.CCR-06-1154

[22] Jolly C and Morimoto RI (2000) Role of the heat shock response and molecular chaperones in oncogenesis and cell death. J Natl Cancer Inst 92, 1564–1572. 11018092 10.1093/jnci/92.19.1564

[23] Han J , Xu X , Qin H , Liu A , Fan Z , Kang L , Fu J , Liu J and Ye Q (2013) The molecular mechanism and potential role of heat shock‐induced p53 protein accumulation. Mol Cell Biochem 378, 161–169. 23456460 10.1007/s11010-013-1607-9

[24] Doong H , Rizzo K , Fang S , Kulpa V , Weissman AM and Kohn EC (2003) CAIR‐1/BAG‐3 abrogates heat shock protein‐70 chaperone complex‐mediated protein degradation: accumulation of poly‐ubiquitinated Hsp90 client proteins. J Biol Chem 278, 28490–28500. 12750378 10.1074/jbc.M209682200

[25] Wang C and Chen J (2003) Phosphorylation and hsp90 binding mediate heat shock stabilization of p53. J Biol Chem 278, 2066–2071. 12427754 10.1074/jbc.M206697200

[26] Miyakoda M , Nakahata K , Suzuki K , Kodama S and Watanabe M (1999) Heat‐induced G1 arrest is dependent on p53 function but not on RB dephosphorylation. Biochem Biophys Res Commun 266, 377–381. 10600511 10.1006/bbrc.1999.1829

